# Supplementary material for: Study of Metabolic Adaptation of Red Yeasts to Waste Animal Fat Substrate
Source: Microorganisms. 2019 Nov 19;7(11):578. doi: 10.3390/microorganisms7110578 (PMC6920810; doi:10.3390/microorganisms7110578)
Supplement: Supplementary file 1 [file microorganisms-07-00578-s001.pdf]

*Supplementary Material*

# Study of metabolic adaptation of red yeasts to waste animal fat substrate

**Martin Szotkowski [1], Dana Byrtusova, Andrea Haronikova, Marie Vysoka, Marek Rapta, Volha Shapaval [2] and Ivana Marova [1,*]**

[1] Faculty of Chemistry, Brno University of Technology, Brno, Czech Republic ; marova@fch.vut.cz
[2] NMBU As, Norway; volha.shapaval@nmbu.no
* Correspondence: marova@fch.vut.cz; Tel.: +420 541 149 419

**Contents:**

Figure S1: Growth curves of some red yeasts

Figure S2: Oil spreading test performed by incubation of sunflower oil with media of red yeast strains cultivated for 96 hours in glucose medium (C/N 13).

Figure S3: Scanning electron microscopy of red yeast cells cultivated on glucose at different C/N ratio

Table S1: Summary of growth and production data of red yeasts cultivated at different media

Table S2: Production of lipids and lipid-soluble metabolites during early stationary phase

Table S3: Summary characteristic of yeast biomass produced on glucose at different C/N ratio

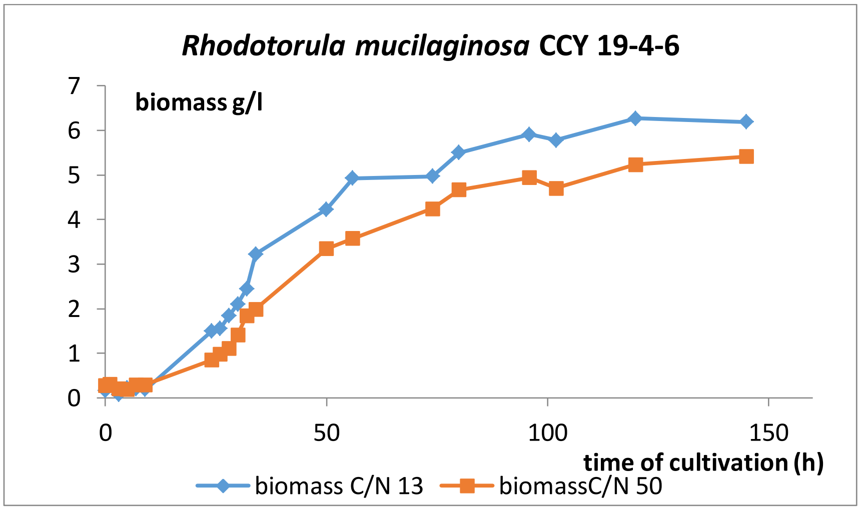


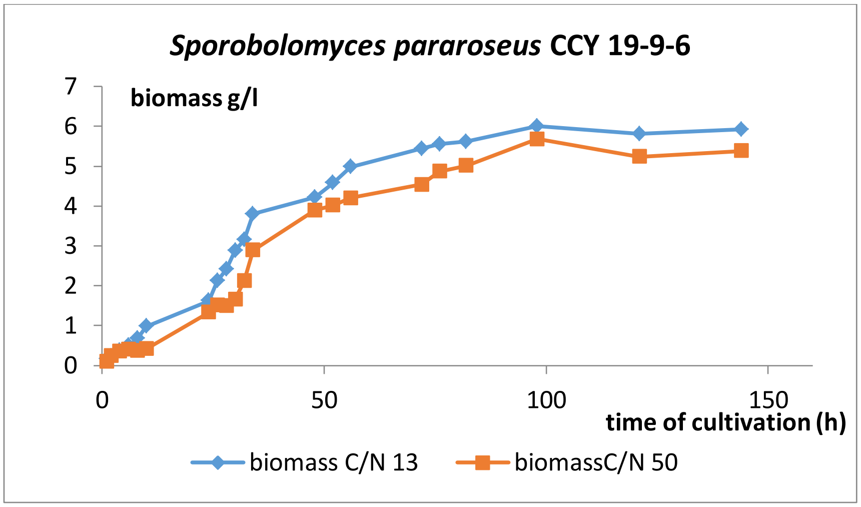


**Figure S1. Growth curves of some red yeasts**

Cultivation experiments were carried out to assess the growth of two yeast strains (*Rhodotorula mucilaginosa* CCY 19-4-6 and *Sporobolomyces pararoseus* CCY 19-9-6 on 2 different media. The shape of growth curves indicates that after 50 – 70 h of growth logarithmic phase is finished and followed by typical prolonged stationary phase with some local maxima and minima of growth as described previously (19). These fluctuations are caused probably by gradual formation and utilization of storage compounds. In both strains biomass formation was slightly lower at C/N 50 when compared with C/N 13. Well-balanced stationary phase was reached after 90 hours in both strains.

Conditions: Cultivation was carried out at 28 °C, pH 5.5, constant stirring (800 rpm) and under permanent light exposure. Cultivations were done in parallel duplicates. Media contained glucose as a C-source. Appropriate amount of glucose (30 g/l for C/N ratio 13 and 99.9 g/l for C/N 50) was added into 1000 ml of water with addition of salts - $KH_2PO_4$ 4 g/l, $(NH_4)_2SO_4$ 4 g/l and $MgSO_4 . 7 H_2O$ 0.696 g/l. The CDW values are means (n = 2).

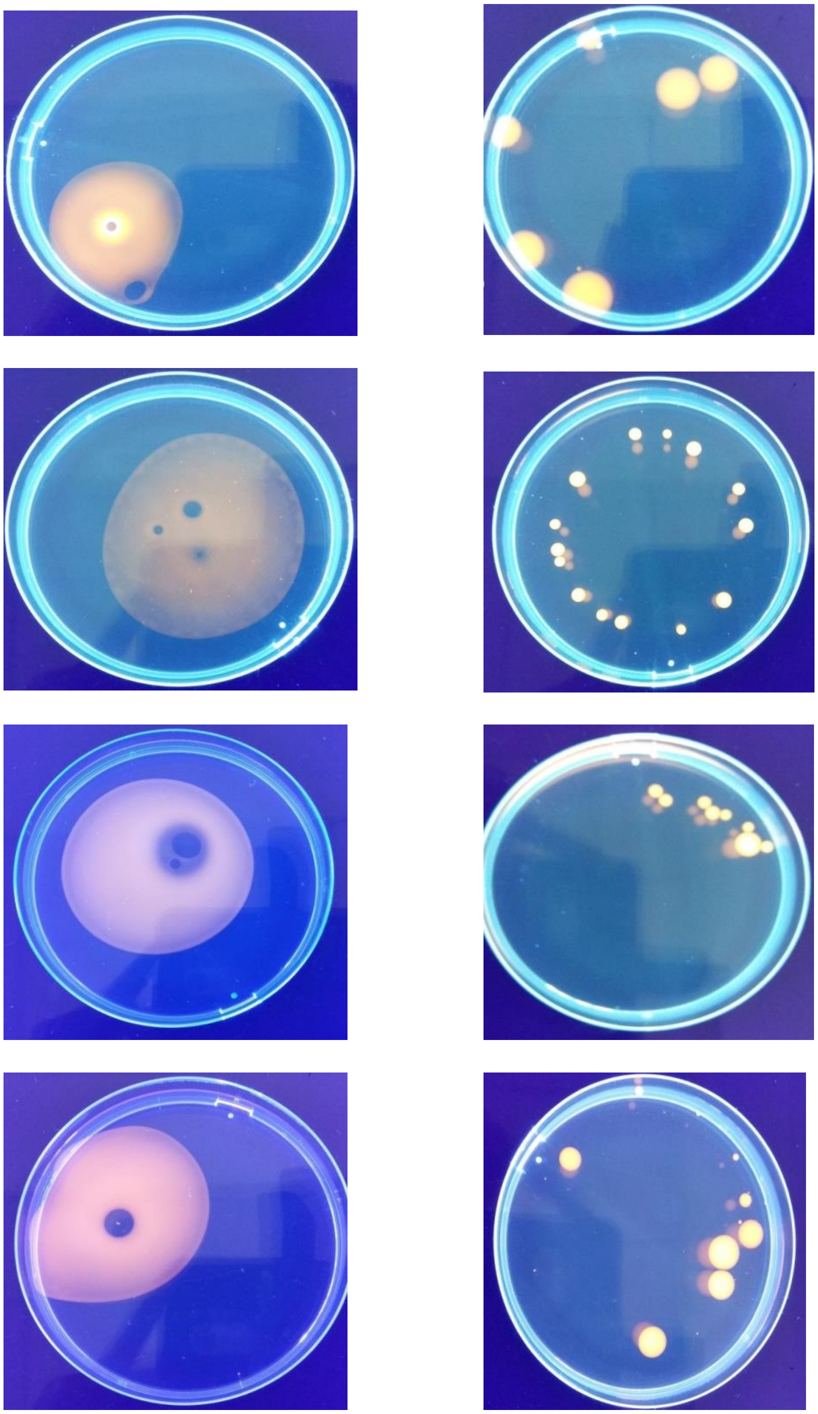

RG

CM

SP

RM

**Figure S2**: Oil spreading test performed by incubation of sunflower oil with media of red yeast strains cultivated for 96 hours in glucose medium (C/N 13).

Left side – control (oil layer on 90 mm Petri dishes). Right side – emulsification effect of yeasts. As a positive control Triton X-100 and negative control water was included into the test. All tested strains exhibited positive emulsification activity.

Abbreviations: CM (*Cystofilobasidium macerans* CCY 10-1-2), RG (*Rhodotorula glutinis* CCY 20-2-26), RM (*Rhodotorula mucilaginosa* CCY 19-4-6) and SP (*Sporobolomyces pararoseus* CCY 19-9-6)

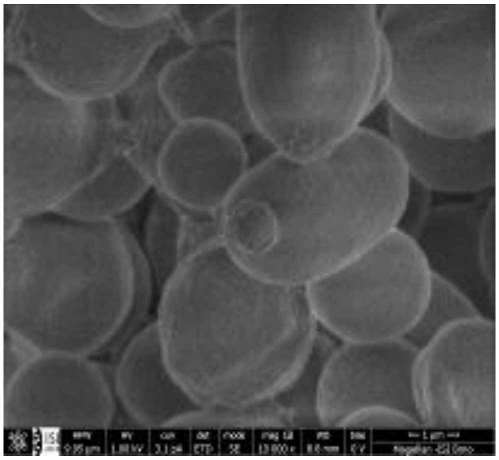

**(a )** *R.glutinis* CCY 20-2-26 C/N 13

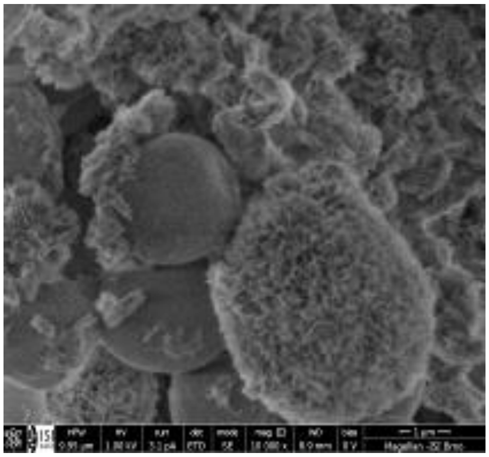

**(b)** *R. glutinis* CCY 20-2-26 C/N 100

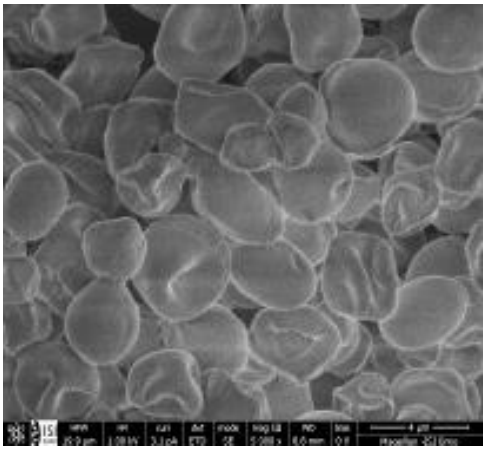

**(c )** *C. macerans* CCY 10-1-2 C/N 13

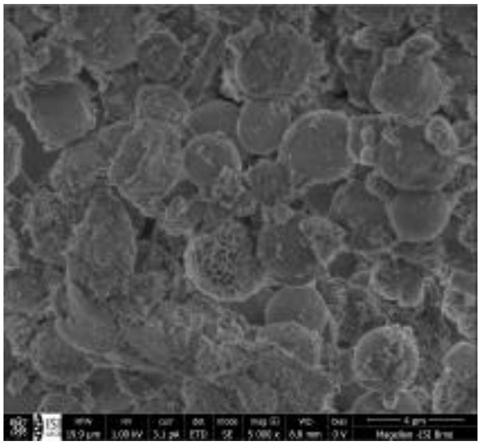

**(d)** *C. macerans* CCY 10-1-2 C/N 100

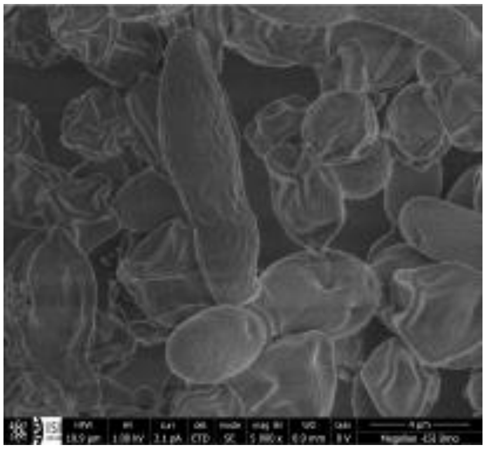

**(e )** *S. pararoseus* CCY 19-9-6 C/N 13

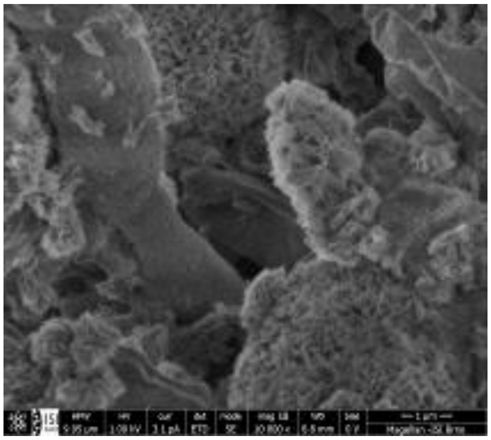

**(f)** *S. pararoseus* CCY 19-9-6 C/N 100

**Figure S3**: Scanning electron microscopy of red yeast cells cultivated on glucose at different C/N ratio

**Table S1:** Summary of growth and production data of red yeasts cultivated at different media

| parameter | medium | strain | | | |
|---|---|---|---|---|---|
| | | Rhodotorula glutinis | Cystofilobasidium macerans | Rhodotorula mucilaginosa | Sporobolomyces pararoseus |
| **biomass** | glucose | 6.63±0.30 | 4.69±0.22 | 4.55±0.29 | 5.88±0.50 |
| **g/L** | glycerol | 5.84±0.28 | 3.90±0.09 | 4.82±0.20 | 3.40±0.14 |
| | crude fat | 4.73±0.19 | 3.82±0.13 | 5.13±0.28 | 7.00±0.39 |
| | emulsified fat | 4.43±0.21 | 2.02±0.07 | 5.45±0.31 | 9.83±0.51 |
| | hydrolyzed fat | 14.40±0.64 | 4.32±0.22 | 9.05±0.85 | 10.08±0.65 |
| **CAR** | glucose | 5.77±0.31 | 2.00±0.39 | 2.20±0.22 | 3.15±0.30 |
| **mg/L** | glycerol | 6.85±0.54 | 4.40±0.65 | 5.15±0.67 | 7.48±0.89 |
| | crude fat | 6.13±0.49 | 5.70±0.76 | 4.00±0.78 | 6.20±0.92 |
| | emulsified fat | 6.50±0.50 | 14.20±0.98 | 8.40±0.84 | 24.50±2.00 |
| | hydrolyzed fat | 12.20±0.98 | 13.30±0.84 | 4.10±0.96 | 18.70±1.65 |
| **ERG** | glucose | 0.47±0.09 | 0.39±0.04 | 0.41±0.06 | 0.49±0.05 |
| **mg/L** | glycerol | 0.62±0.11 | 0.37±0.04 | 0.68±0.08 | 0.61±0.05 |
| | crude fat | 0.50±0.08 | 0.48±0.06 | 0.59±0.08 | 0.40±0.09 |
| | emulsified fat | 0.37±0.09 | 0.58±0.09 | 0.52±0.11 | 0.56±0.09 |
| | hydrolyzed fat | 0.48±0.11 | 0.61±0.09 | 0.39±0.12 | 0.62±0.09 |
| **UBI** | glucose | 1.70±0.31 | 0.90±0.02 | 1.85±0.22 | 4.35±0.34 |
| **mg/L** | glycerol | 4.40±0.56 | 2.30±0.30 | 6.82±0.56 | 3.10±0.22 |
| | crude fat | 7.22±0.82 | 3.80±0.53 | 5.30±0.98 | 4.30±0.24 |
| | emulsified fat | 2.50±0.76 | 6.50±0.98 | 6.70±0.87 | 7.20±0.76 |
| | hydrolyzed fat | 4.20±0.54 | 7.00±0.67 | 3.30±0.85 | 11.30±0.78 |
| **LIPIDS** | glucose | 20.70±1.19 | 21.60±1.12 | 19.90±1.43 | 25.80±1.74 |
| % | glycerol | 11.50±0.98 | 10.80±0.67 | 8.70±1.01 | 7.30±1.09 |
| | crude fat | 14.00±1.45 | 14.70±1.98 | 26.50±2.12 | 48.00±4.52 |
| | emulsified fat | 12.20±1.25 | 14.00±1.65 | 39.30±2.21 | 54.50±4.65 |
| | hydrolyzed fat | 22.50±2.82 | 42.10±4.11 | 45.40±2.90 | 48.50±3.98 |

Strains: CM (*Cystofilobasidium macerans* CCY 10-1-2), RG (*Rhodotorula glutinis* CCY 20-2-26), RM (*Rhodotorula mucilaginosa* CCY 19-4-6) and SP (*Sporobolomyces pararoseus* CCY 19-9-6).

Metabolites: CAR (carotenoids m/l); ERG (ergosterol mg/l); UBI (ubiquinone mg/l);

**Table S2.** Production of lipids and lipid-soluble metabolites during early stationary phase

| | Time (hours) | glucose | | | | glycerol | | | | Crude fat | | | |
|---|---|---|---|---|---|---|---|---|---|---|---|---|---|
| | | CAR | ERG | UBI | %LIP | CAR | ERG | UBI | %LIP | CAR | ERG | UBI | %LIP |
| **RG** | 60 | 2.6 | 0.30 | 1.5 | 13.5 | 6.3 | 0.38 | 1.9 | 9.8 | 3.6 | 0.32 | 5.8 | 12.8 |
| | 72 | 4.7 | 0.21 | 2.1 | 17.1 | 2.0 | 0.17 | 0.9 | 12.0 | 3.9 | 0.47 | 3.6 | 14.1 |
| | 78 | 5.3 | 0.53 | 2.8 | 17.2 | 3.8 | 0.24 | 1.4 | 11.6 | 4.0 | 0.47 | 5.7 | 15.0 |
| | 84 | 5.4 | 0.48 | 1.4 | 20.0 | 5.9 | 0.31 | 1.9 | 11.4 | 3.9 | 0.45 | 5.1 | 13.9 |
| | 96 | 5.8 | 0.47 | 1.7 | 20.7 | 6.8 | 0.62 | 4.4 | 11.5 | 6.1 | 0.50 | 7.2 | 14.0 |
| **CM** | 60 | 1.9 | 0.25 | 0.7 | 13.9 | 3.5 | 0.28 | 1.2 | 10.0 | 5.2 | 0.42 | 3.2 | 16.8 |
| | 72 | 2.0 | 0.27 | 0.8 | 15.3 | 5.9 | 0.38 | 5.9 | 9.6 | 4.9 | 0.40 | 3.4 | 16.0 |
| | 78 | 2.1 | 0,28 | 1.0 | 15.4 | 4.3 | 0.36 | 2.4 | 9.7 | 7.2 | 0.55 | 3.4 | 15.4 |
| | 84 | 2.1 | 0.29 | 1.5 | 17.5 | 5.2 | 0.44 | 3.2 | 9.6 | 5.4 | 0.58 | 3.3 | 15.8 |
| | 96 | 2.0 | 0.39 | 0.9 | 21.6 | 4.4 | 0.37 | 2.3 | 10.8 | 5.7 | 0.48 | 3.8 | 14.7 |
| **RM** | 60 | 2.6 | 0.43 | 0.7 | 17.8 | 1.9 | 0.37 | 0.7 | 9.9 | 3.5 | 0.39 | 3.6 | 27.3 |
| | 72 | 2.7 | 0.40 | 0.7 | 15.6 | 3.0 | 0.60 | 2.6 | 7.5 | 4.0 | 0.49 | 3.9 | 29.8 |
| | 78 | 3.3 | 0.38 | 1.9 | 19.4 | 2.9 | 0.53 | 2.8 | 8.6 | 3.9 | 0.40 | 4.0 | 26.2 |
| | 84 | 3.4 | 0.42 | 2.1 | 18.4 | 2.3 | 0.44 | 1.9 | 8.4 | 4.6 | 0.60 | 5.9 | 26.8 |
| | 96 | 2.2 | 0.41 | 1.9 | 19.9 | 4.2 | 0.58 | 2.8 | 8.7 | 4.0 | 0.59 | 5.3 | 26.5 |
| **SP** | 60 | 5.0 | 0.62 | 3.9 | 23.0 | 4.8 | 0.37 | 5.3 | 11.5 | 8.7 | 0.84 | 18.6 | 46.5 |
| | 72 | 5.1 | 0.38 | 4.6 | 22.9 | 6.3 | 0.43 | 2.8 | 11.7 | 13.4 | 0.96 | 17.5 | 50.6 |
| | 78 | 5.8 | 0.49 | 7.2 | 24.1 | 6.9 | 0.57 | 2.7 | 8.8 | 10.1 | 0.74 | 9.8 | 48.2 |
| | 84 | 6.1 | 0.47 | 9.2 | 24.2 | 4.0 | 0.34 | 2.0 | 9.2 | 10.4 | 0.69 | 8.7 | 50.0 |
| | 96 | 5.2 | 0.49 | 8.4 | 25.8 | 7.5 | 0.61 | 3.1 | 7.3 | 9.2 | 0.70 | 8.3 | 48.0 |

Metabolite concentrations in periodically taken samples cultivated to early stationary phase (according to point (b) in Materials and Methods, Paragraph 2.3.2. Values are means (n = 3). Because of illustrative character of these data, ± SD are not introduced. Values of SD were in the range of 2 – 10 % for metabolites and 0 – 5% for biomass determination, similarly to data introduced in Table S1.

Strains: CM (*Cystofilobasidium macerans* CCY 10-1-2), RG (*Rhodotorula glutinis* CCY 20-2-26), RM (*Rhodotorula mucilaginosa* CCY 19-4-6) and SP (*Sporobolomyces pararoseus* CCY 19-9-6).

Metabolites: CAR (carotenoids m/l); ERG (ergosterol mg/l); UBI (ubiquinone mg/l); %LIP (lipids; % in g of CDW)

**Table S3**: Summary characteristic of yeast biomass produced on glucose at different C/N ratio

| | **C/N 13** | | | | | **C/N 25** | | | | |
|---|---|---|---|---|---|---|---|---|---|---|
| **strain** | **Glucans** % | **lipids** % | **CAR** % | **ERG** % | **UBI** % | **Glucans** % | **lipids** % | **CAR** % | **ERG** % | **UBI** % |
| **RG** | 23.18 | 9.13 | 0.087 | 0.007 | 0.026 | 23.80 | 32.85 | 0.024 | 0.008 | 0.056 |
| **CM** | 26.15 | 5.95 | 0.043 | 0.008 | 0.019 | 23.61 | 35.23 | 0.063 | 0.010 | 0.016 |
| **RM** | 17.52 | 12.72 | 0.048 | 0.009 | 0.041 | 14.60 | 34.19 | 0.052 | 0.011 | 0.028 |
| **SP** | 14.30 | 11.08 | 0.054 | 0.008 | 0.074 | 16.87 | 30.86 | 0.074 | 0.003 | 0.044 |

| | **C/N 50** | | | | | **C/N 100** | | | | |
|---|---|---|---|---|---|---|---|---|---|---|
| **strain** | **Glucans** % | **lipids** % | **CAR** % | **ERG** % | **UBI** % | **Glucans** % | **lipids** % | **CAR** % | **ERG** % | **UBI** % |
| **RG** | 21.05 | 37.32 | 0.030 | 0.004 | 0.020 | 20.35 | 36.52 | 0.026 | 0.003 | 0.013 |
| **CM** | 18.53 | 46.52 | 0.028 | 0.005 | 0.012 | 16.32 | 47.27 | 0.007 | 0.005 | 0.007 |
| **RM** | 15.54 | 34.23 | 0.070 | 0.008 | 0.032 | 15.99 | 33.26 | 0.008 | 0.007 | 0.008 |
| **SP** | 15.58 | 39.36 | 0.074 | 0.008 | 0.033 | 14.73 | 39.12 | 0.014 | 0.002 | 0.010 |

For this comparison glucan concentrations (values are means (n=2)) are introduced as described in Table 2. Average total lipid values (percentage of CDW) were used (without SD) as introduced in Table 3. Average values of percentage of metabolites in biomass were calculated from the values of production expressed as milligram per gram of CDW.

Strains: CM (*Cystofilobasidium macerans* CCY 10-1-2), RG (*Rhodotorula glutinis* CCY 20-2-26), RM (*Rhodotorula mucilaginosa* CCY 19-4-6) and SP (*Sporobolomyces pararoseus* CCY 19-9-6).

Abbreviations: CAR (total carotenoids), ERG (ergosterol), UBI (ubiquinone)
